# Biophilia and Biophobia as Emotional Attribution to Nature in Children of 5 Years Old

**DOI:** 10.3389/fpsyg.2020.00511

**Published:** 2020-03-20

**Authors:** Pablo Olivos-Jara, Raquel Segura-Fernández, Cristina Rubio-Pérez, Beatriz Felipe-García

**Affiliations:** ^1^Department of Psychology, School of Labor Relations and Human Resources, University of Castilla-La Mancha, Albacete, Spain; ^2^Department of Pedagogy, School of Education, University of Castilla-La Mancha, Albacete, Spain; ^3^CRA Río Mundo, Agramón, Albacete, Spain; ^4^Molinicos Town Hall, Women’s Center, Albacete, Spain

**Keywords:** biophilia, biophobia, connectedness, nature, emotion, emoji, children

## Abstract

**Introduction:**

Connectedness to nature is a concept that reflects the emotional relationship between the self and the natural environment, based on the theory of biophilia, the innate predisposition to the natural environment. However, the biophobic component has largely been ignored, despite, given its adaptive functional role, being an essential part of the construct. If there is a phylogenetic component underlying nature connectedness, biophilic, and/or biophobic, there should be evidence of this record from early childhood. The main aim of this study is therefore to describe the emotional attributions identified in 5 years old.

**Methodology:**

Two studies were conducted. In the first, 94 children expressed their concept of nature and made basic emotional attributions to a set of 30 images of natural, using a software designed for the study. In the second, 39 children repeated the procedure and provided explanations for their responses.

**Results:**

The main results show that, in general, children use both positive and negative emotions, which may be related to a three-dimensional model of emotional attributions to nature. The most widely attributed emotion is happiness. However, fear is the second most common attribution. The role of happiness could be explained by a feeling of security and familiarity, while the importance of fear in nature could show an adaptive response of the fear of wild nature in children. This interpretation could be confirmed when analyzing specifically the emotional attributions, classifying the images according to biological and ecosystemic criteria. Thus, for example, more emotional attributions are explained by the “pleasantness” attributed to primary producers and landscapes (e.g., flora), versus attributions of “harm” to the images of secondary and tertiary consumers (e.g., hunters).

**Conclusion:**

These results provide evidence in favor of a didactic procedure to study emotional attributions to images of nature in preschool children. They suggest the incorporation of biophobia as an important adaptive factor in connectedness to nature and a tripartite emotional hypothesis based on the valences of the attributed emotions.

## Introduction

Since Clayton (2003) and Mayer and Frantz (2004) proposed the concepts of environmental identity and connectedness to nature, several studies have highlighted the link between the environment and people through self or identity. These concepts include the self-perceived affective relationship of the interconnection between the self and the natural environment. However, unlike environmental identity, the original approach to connectedness (Mayer and Frantz, 2004; Schultz et al., 2004) was founded on phylogenetic arguments, drawing on the theory of Biophilia. Originally proposed by Wilson (1984), this refers to an innate and positive human predisposition of affiliation to the natural environment, which allows the human being to experience benefits that, according to its author, facilitated the development, adaptation and survival of human beings. However, the biophobic component of connectedness with nature has largely been ignored, despite, given its adaptive functional role, being an essential part of the construct. This lack in the conceptual field faces the present study.

Although many alternative measures have been developed to the connectedness to nature scale originally proposed by Mayer and Frantz (e.g., Kals et al., 1999; Schultz, 2001; Dutcher et al., 2007; Davis et al., 2009; Nisbet et al., 2009; Pasca et al., 2017), this construct remains one of the most widely used in studies on the relationship between self and nature, with these works always adopting a biophilic perspective (Brügger et al., 2011; Tam, 2013; Olivos and Clayton, 2017). Another important characteristic of connectedness is its emotional component (Kals et al., 1999; Mayer et al., 2009; Howell et al., 2011; Cervinka et al., 2012), which has been studied in relation to subjective well-being (Gillis and Gatersleben, 2015; Olivos and Clayton, 2017).

Some authors, such as Perrin and Benassi (2009) have argued that the connectedness to nature scale fails to measure an emotional component. However, the study of well-being in relation to the environment has a solid empirical basis, which has been approached from different conceptualizations. The most frequent approach to connectedness has been the study of hedonic well-being – also called subjective or emotional well-being – based on the registration of positive emotions as a result of direct contact with natural stimuli (Saraglou et al., 2008; Weinstein et al., 2009; Nisbet et al., 2011). The results usually point to the experience of positive sensations after direct exposure to nature (Mayer et al., 2009), to residence near green environments (Astell-Burt et al., 2014; Fattorini et al., 2017), even after mere exposure to images (Falsten, 2014; Mena et al., 2020) or the evocation of natural landscapes (Hinds and Sparks, 2011). Some of these studies show the mediating role of connectedness between environmental stimuli and well-being, in such a way that nature has a buffering effect for stress reduction, improves attention tasks, promotes positive social behaviors, pro-environmental behaviors, connectedness to nature, and in short, improves quality of life (Mayer et al., 2009; Corraliza and Collado, 2011; Hoot and Riedman, 2011; Nisbet and Zelenski, 2011; Carrus et al., 2012; Myers, 2012; Howell et al., 2013; Collado and Corraliza, 2016; Collado and Staats, 2016). However, other authors have observed that an adequate prior connectedness feature is not required to be effective in improving emotional well-being through experiences of contact with natural environments (Passmore and Howell, 2014).

Considering, then, the emotional content of biophilia, connectedness with nature would act as a kind of phylogenetically oriented guide, favoring the search for the individual, material and emotional benefits, through contact with the environment. Therefore, if the phylogenetic relationship of human beings with their environment must be resolved favorably toward survival, then a negative phylogenetic disposition, of a biophobic type, must be expected, consisting of emotions that allow an alert, safe reaction to certain threats present in nature.

Biophobia has been considered by other authors, who describe it as the feeling of fear or rejection of natural elements with an adaptive purpose (Ulrich, 1993; Orians, 1998). It produces emotional reactions of negative valence in reaction to certain natural stimuli (such as a dangerous animal or a natural catastrophe) with the aim of promoting protective, rejection or withdrawal behaviors to avoid harm (Koole and Van den Berg, 2005). In this line, Hand et al. (2017) point out that children do not behave as predicted by the biophilic hypothesis, because, in some cases, they avoid biodiverse spaces due to their producing negative emotions.

Although biophobia is an unexplored field in Environmental Psychology, due to the bias imposed by positive psychology on the study of well-being (Brown et al., 2018; Olivos and Ernst, 2018), some studies have highlighted affective ambivalence effects, such as anxiety or isolation responses, after contact with certain natural environments (Hinds and Sparks, 2011; Davis and Gatersleben, 2013; Gatersleben and Andrews, 2013). Biophobia may activate other phylogenetic components of connectedness and, like biophilia, may also be subject to processes of sociocultural symbolization and epigenetic adaptation.

The scientific literature lacks instruments to measure negative affectivity as a dimension with positive adaptive effects for individuals. There exist a few scales developed *ad hoc* to record negative emotions (Hinds and Sparks, 2011; Davis and Gatersleben, 2013), and other scales that measure negative emotions versus positive emotions in the well-being concept (for example, PANAS, Watson et al., 1988; SPANE, Diener et al., 2010; ZIPERS, Zuckerman, 1977) but their interpretation is usually negatively stigmatized as undesirable response. There is, therefore, a need to develop a procedure for positive and negative emotion measurement, according to the effects that the perception of nature may cause, conceived for the description of biophilia and biophobia, interpretable as adaptive mechanisms.

## Children, Emotion, and Nature

Studies on experiences of contact with nature in young and adult populations have shown the importance of childhood memories in the impressions evoked by these experiences (Thomashow, 1995; Schroeder, 2007; Bartos, 2013; Olivos et al., 2013; Mena et al., 2020). The results suggest that the significance of the environment depends on the emotional impact of a person’s early experiences. Thus, it is of key importance to determine how children interact with the environment and the emotions evoked by contact with natural stimuli.

However, most studies to assess children’s pro-environmental attitudes and behaviors, on contact with nature, have been conducted as part of structured environmental education programs, with samples of children aged above 8 years. Most of the studies were conducted using pencil-and-paper questionnaires (e.g., Kanh and Kellert, 2002; Kals and Ittner, 2003; Wells and Evans, 2003; Wells and Lekies, 2006; Maller, 2009; Bruni and Schultz, 2010; Van den Berg and Van den Berg, 2010; Carrus et al., 2012; Collado et al., 2013; Corraliza et al., 2013; Collado and Corraliza, 2016).

One approach to the biophilic and biophobic principles of nature connectedness consists of observing the emotional components it induces in early childhood, due to children’s lower exposure to socialized symbolic content compared to adolescents, young persons and adults.

During the first year of life, children experience primary emotions and dichotomous models of relational interaction (laughing or crying, happiness or sadness), mainly associated with internal physiological states (hunger, sleepiness, etc.). Happiness and anger are emotions that infants can recognize in others after just 1 month of life. However, it is from the age of 4–8 months when they begin to differentiate between them, improving their expressive capacity, and adding reactions of surprise. It is after this stage that infants begin to distinguish between positive and negative emotions and expressions of fear and guilt emerge (Sprung et al., 2015). Between the first and third year of life, empathy begins to develop and basic emotions are consolidated, which infants are now able to imitate (Jones and Mize, 2016). At 4 years, due to language development resulting from the linguistic and conceptual acquisition of graphic expression (Remplein, 1966; Bomfim, 2003; Myers, 2012), their conceptual repertoire increases, being able to recognize and name emotions (Segura and Arcas, 2004; Cejudo, 2015). Hence, emotional awareness develops (understanding what you feel and why), although this is still a stage of extremes (great sadness or joy). Emotion regulation begins between 4 and 5 years of age, bolstered by emergence of symbolic play. Nonetheless, the predomination of egocentricity continues until 6 years, when contact and understanding of the social world is enhanced (Sprung et al., 2015).

Children aged between 2 and 5 years begin to have a sense of self and control of their identity, forming an initial sense of connection to the world, which, if developed securely, creates a bond generating well-being and emotional attachment to the natural world (Barraza, 1998; Myers, 2012; Green et al., 2016; Tugurian and Carrier, 2017). Furthermore, at this age, children have not initiated the formal learning of reading and writing skills, which constitutes one of the most powerful socializing influences on the structure of thought (Vygotsky, 1934; Habermas, 1984; Langer and Applebee, 1985; Palinscar, 1998). As the process of representing emotions begins before the acquisition of written expression, images, at an early age, offer the opportunity for greater expression of emotions and feelings, being a more favorable means of expression for children, even for those with reduced social interaction skills (Bomfim, 2003; Ulker, 2012). Hence, it is possible to study the recognition of basic emotions in children using photos (Nelson and Rusell, 2016; Brechet, 2017), drawings (Brechet, 2017), and storytelling (Widen et al., 2015). Some studies have reported better effects of these resources in 5 years old than in older children (Brechet, 2017).

In light of the above, it is important to study connectedness to nature based on emotional attributions toward natural environments in early childhood, in order to delve into the biophilic and biophobic components related to the characteristic elements of the natural world. The aim, then, of the present study is to describe the emotional attributions toward environmental stimuli made by 5 years old boys and girls.

## Methodology

This work involved conducting two studies, the methodological characteristics of which are described below.

## Study 1

### Participants

Initially, 98 participants aged under 6 years were selected using convenience sampling. These were all enrolled in the 5 years old pre-school classes at Benjamín Palencia and Cristóbal Colón Public Infant and Primary Schools in the city of Albacete, Spain. Of these 98 children, 41.2% were girls. A small number of the children had special educational needs in the form of language difficulties and impaired cognitive development and understanding and were consequently excluded from the sample analyzed, which finally comprised 94 children (*M* = 5.7 years; *SD* = 0.6).

### Instrument and Procedure

Taking into account the aims and hypotheses and the differences in the stages of cognitive and moral development compared to older children (Mestre et al., 2011; Lemos, 2013), a measurement procedure was designed to assess the 5 years old emotional attributions toward natural environments.

**FIGURE 1:**
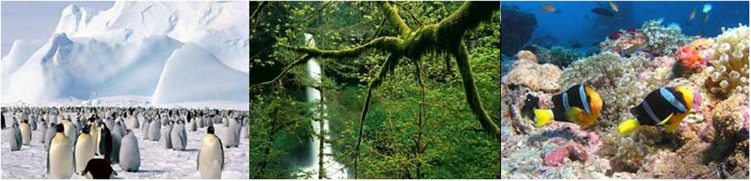
Some of the images of natural environments used in the emotional attribution test (Sánchez et al., 2012).

As the emotion attribution procedure was to be conducted based on exposure to images, the first step was to select such images (Figure 1). We worked with the images of natural environments used in the Environmental Preference Scale (EPS) designed by Sánchez et al. (2012). We choose those images because they formed part of an implicit association test (IAT), which procedure includes a strict prior evaluation (valence, familiarity, activation) as IAT studies demand (De Houwer, 2003; Ruiz and Ortiz, 2006; Verges and Duffy, 2010; Olivos and Aragonés, 2013).

The stimuli were presented in random order, and trial images were included at the beginning to check for correct identification of the icons representing the emotions.

We first conducted a pilot test with the participation of 10 children, 70% boys, with a mean age of 4.5 years (*SD* = 0.47), to check images were identified correctly and the response procedure was understood. The children evaluated the images using emoticons to attribute four basic emotions posited in the literature (Harris, 1994; Davidson, 2006): happiness, sadness, anger and fear. Emoticons are increasingly being used as response protocols in satisfaction evaluations in different settings (shopping malls, airports, public services, etc.) and studies (e.g., Gallo et al., 2017; Schouteten et al., 2018), while also being a common element of communication systems in both children and adults. The results of this trial indicated that, despite appropriately using the emoticons to attribute emotions to the images, the children’s explanations during the attributional process suggested they were able to use at least another emotion within their repertoire.

**FIGURE 2:**
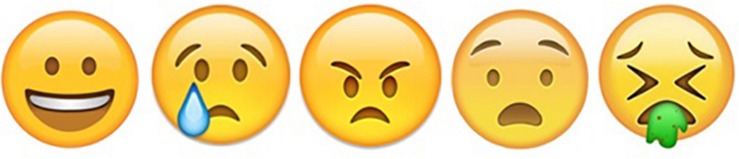
Emoticons from the response scale, emoticons reflecting happiness, sadness, anger, fear and disgust, in that order.

Taking the above into account and the fact there exists no consensus on the predominant set of basic emotions in early childhood, in this first study, we decided to follow the basic emotions theory espoused by Ekman (1999), and used five emoticons (Figure 2): happiness, sadness, anger, fear, and disgust.

To validate the emoticons, an inter-rater reliability analysis, with three expert judges, was conducted to determine whether the emoticons adequately represented the associated emotions. The mean Kappa coefficient was 0.350, equivalent to “correct” (Dubé, 2008). Moreover, 90% of the children correctly associated the emotions represented in the trial images with the corresponding emoticons.

**FIGURE 3:**
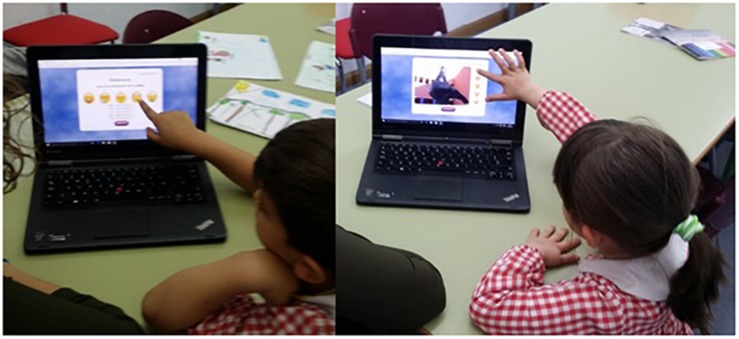
Images of a boy and a girl using the application on the touch screen laptop (written informed consent for publishing images available).

To present the images and record the responses, we developed a web-based application using HTML language, with PHP server language. To run the application, an Apache server was used as well as an MYSQL database to store the information generated, which was subsequently exported to an Excel spreadsheet. The applications were partly developed by a trained survey expert, who used a Lenovo Yoga laptop computer, with Windows 10 business edition, Intel Core i5 de 1.60 GHz processor and a touch screen (Figure 3).

We contacted the school principals and the class teachers of the groups of 5 years old, with whom we subsequently held an informational meeting. Once they agreed to collaborate, we distributed authorization letters to parents and guardians, with an informed consent form and a short family questionnaire to collect sociodemographic data.

The procedure was administered individually, with each session lasting an average of 22 min. The first thing the children did was to answer an open question on what nature meant to them. The next stage was conducted in front of the computer (game), and was divided into three parts. In the first part, the children were required to associate the name of each emotion with its corresponding emoticon. This served to check the validity of the emotional pictographic scale and for the children to become acquainted with the response protocol. The second part was composed of five trial images, and the third consisted of the presentation of the images included in the study.

A lexicographic analysis was conducted on the open responses on the concept of nature using the “open coding” procedure (Strauss and Corbin, 1994), to identify the children’s ideas on the concept of nature. This technique has been used in other environmental psychology studies (Schroeder, 2007; Mena et al., 2020). It involves the quantitative analysis of certain word patterns within a comprehensive documentary corpus, organized into categories (labels).

The data analysis was conducted using SPSS24 software, with frequency and simple correspondence analysis to describe the relationships between the nominal variables in a correspondence table in a low-dimensional space, to describe the new dimensional categories build from the weighed intersected position between those variables. For the adequate interpretation of the correspondence analyses, we used both quantitative and qualitative criteria. First, to retain the number of dimensions resulted we used the more common rules which recommend that their added inertia represents as a minimum of 70% (Higgs, 1991). Additionally, we retain dimensions with eigenvalues over 10% (Nagpaul, 1999). After that, we analyze them taking in to account theoretical assumptions and objectives, following a whole comprehension of the dimensions and the axis resulted from the extremes contents involve.

### Results

#### Concept of Nature

The analysis of the responses on the concept of nature resulted in a table with 14 labels from a total corpus of 316 words. The largest category was “vegetation,” accounting for 31.28% of mentions, with the words including flowers and trees. The next largest category was “animals,” which included 32.29% of the mentions. This category, however, was subdivided into “invertebrates” (16.76%), “domestic animals” (9.50%), “woodland animals” (2.52%), “wild animals” (2.24%), and “reptiles” (1.27%).

The next category was “natural processes” (10.42%), including seasons, such as spring; expressions, such as cycles; and actions, such as living or coming out, referring to flowers, the sun, butterflies, etc.

The following label was “inanimate nature” (6.34%, e.g., water, rainbow), followed by “celestial bodies” (4.10%, e.g., star, moon, sun) and “built environment” (2.87%, e.g., cars, schools, motorbikes). These last two categories are interesting in the children’s concept of nature, as the first refers to indirectly experienced natural elements, and the second to non-natural elements.

Finally, accounting for lower percentages, are the labels of “well-being” (3.18%, e.g., happy, peaceful, having a good time), “esthetic emotion” (2.89%, e.g., pretty, cool, smell good), “human beings” (1.59%, e.g., humans, people), and “fantasy” (0.64%, e.g., dinosaur, vampire).

#### Emotional Attributions

We then performed a frequency analysis on the number of times each emoticon was selected. Happiness was also the most popular choice of emotion for the natural environment images (47.27%), when they featured fish, penguins and landscapes of fields with yellow flowers. Fear was the second most frequently chosen emotion for natural environments (27.62%), in response to images of a shark and a snake. The third most frequently elicited emotion for nature environments was disgust (11.10%), elicited by images of insects. The fourth most frequently chosen emotion was sadness (8.30%), in response to images of a cactus and a scorpion. Finally, anger (5.71%) was the lowest ranked emotion, in response to a cactus and a forest with smoke.

**FIGURE 4:**
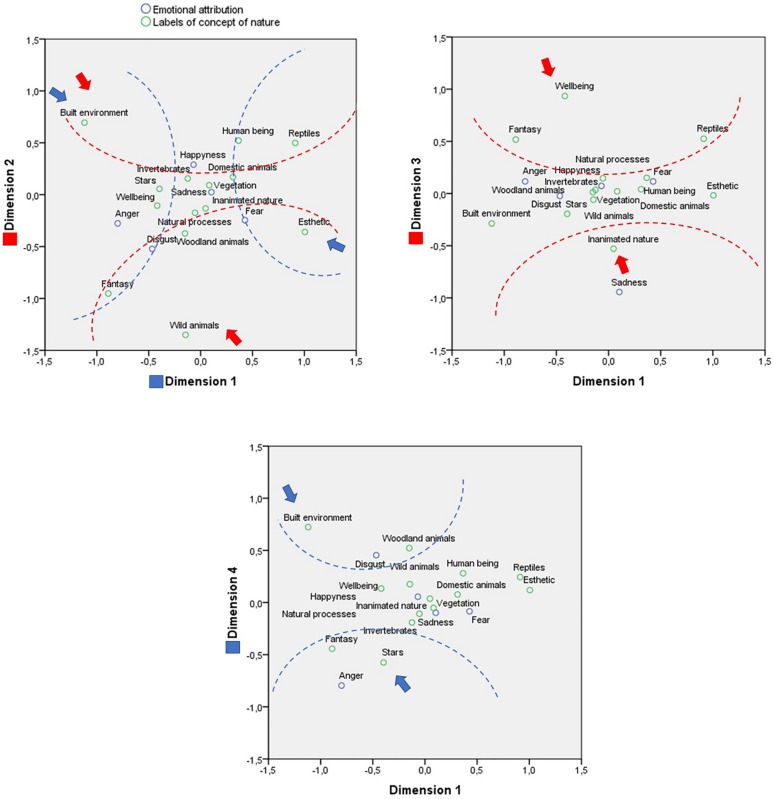
Correspondence analysis between concept of nature and emotional attribution in images of a natural environment.

Next, a simple correspondence analysis was conducted between the emotional attributions and the labels of the concept of nature, according to the environment type (Figure 4). We obtained statistically significant coefficients in the observation of both the row points (χ^2^ = 202.482; *p* < 0.001), referring to the labels of the concept of nature, and the column points, referring to the emotions. This reflects the existence of a relationship between the frequency of the nature labels and the emotions attributed to the images of nature environment.

To interpret theses data, it is advisable to take into account the categories of content and emotions with the greatest explanatory power, as there are few truly clear associations. The analysis of the relative contributions to the examination of the row points, for the attributions toward both types of environment, reveals high representativeness, with most of the values close to one (>0.800) in the dimensions.

In the attributions (Table 1), four dimensions were identified that together explain 100% of the variance. The first dimension explains 42.2%. In one extreme of the first dimension we find “built environment,” “fantasy,” “well-being,” and “celestial bodies,” associated with anger; and in the other extreme, we find the labels of “esthetic emotion,” “reptiles,” “human beings,” and “domestic animals,” associated with fear. In other words, the first dimension discriminates between predominately subjective elements with negative emotional valence and living beings associated with fear.

**TABLE 1:** Symmetrical normalization of row and column points of the correspondence analysis between concept of nature and emotional attribution in images of a natural environment.

	Mass					Inertia	Contribution								
							
		Score in dimension					Of point to inertia of dimension				Of dimension to inertia of point				
					
		1	2	3	4		1	2	3	4	1	2	3	4	Total
**Overview row points: labels of concept of nature**															
Vegetation	0.257	0.082	0.091	0.021	–0.052	0.000	0.015	0.023	0.001	0.011	0.448	0.436	0.019	0.098	1.0
Inanimated nature	0.113	0.048	–0.133	–0.528	0.038	0.003	0.002	0.022	0.423	0.003	0.011	0.070	0.915	0.004	1.0
Stars	0.050	–0.397	0.056	–0.195	–0.575	0.002	0.069	0.002	0.025	0.266	0.432	0.007	0.068	0.493	1.0
Natural processes	0.113	–0.053	–0.174	0.146	–0.108	0.001	0.003	0.038	0.032	0.021	0.060	0.512	0.295	0.133	1.0
Domestic animals	0.108	0.312	0.171	0.041	0.077	0.002	0.093	0.035	0.002	0.010	0.778	0.187	0.009	0.026	1.0
Wild animals	0.018	–0.145	–1.351	–0.059	0.175	0.003	0.003	0.363	0.001	0.009	0.014	0.973	0.002	0.011	1.0
Country animals	0.063	–0.151	–0.373	0.013	0.522	0.002	0.013	0.097	0.000	0.279	0.080	0.395	0.000	0.524	1.0
Invertebrates	0.145	–0.125	0.156	0.035	–0.191	0.001	0.020	0.039	0.002	0.085	0.279	0.350	0.014	0.356	1.0
Reptiles	0.014	0.912	0.498	0.525	0.244	0.002	0.099	0.037	0.050	0.013	0.669	0.159	0.146	0.026	1.0
Esthetic	0.027	1.005	–0.360	–0.018	0.119	0.003	0.241	0.039	0.000	0.006	0.901	0.092	0.000	0.007	1.0
Wellbeing	0.034	–0.418	–0.105	0.934	0.134	0.003	0.052	0.004	0.393	0.010	0.228	0.012	0.747	0.013	1.0
Human being	0.023	0.366	0.521	0.151	0.280	0.001	0.027	0.068	0.007	0.029	0.327	0.531	0.037	0.105	1.0
Built environment	0.027	–1.119	0.694	–0.287	0.723	0.006	0.299	0.144	0.030	0.230	0.634	0.195	0.027	0.144	1.0
Fantasy	0.009	–0.890	–0.953	0.517	–0.443	0.002	0.063	0.090	0.032	0.029	0.439	0.404	0.098	0.059	1.0
**Overview column points: emotional attribution**															
Happyness	0.480	–0.068	0.289	0.073	0.055	0.004	0.020	0.442	0.035	0.024	0.060	0.872	0.046	0.022	1.0
Sadness	0.076	0.104	0.024	–0.944	–0.099	0.005	0.007	0.000	0.905	0.012	0.018	0.001	0.973	0.009	1.0
Anger	0.056	–0.799	–0.276	0.116	–0.796	0.007	0.315	0.047	0.010	0.572	0.606	0.058	0.008	0.327	1.0
Fear	0.280	0.427	–0.245	0.115	–0.084	0.008	0.451	0.186	0.050	0.032	0.750	0.198	0.036	0.016	1.0
Disgust	0.108	–0.467	–0.523	–0.023	0.454	0.007	0.207	0.325	0.001	0.359	0.397	0.399	0.001	0.204	1.0

The second dimension explains 27.1% of the variance. At one extreme are situated “wild animals,” “fantasy,” “woodland animals,” and “esthetic emotion,” associated with disgust; and at the other extreme “built environment,” “human beings,” and “reptiles,” which, although they have no defined weighting, are close to happiness. This implies that the second dimension discriminates between a threatening natural environment, and living beings in an urban environment (as pets) with a positive emotional valence.

The third dimension explains 18.2% of the variance. At one extreme we find only “inanimate nature” associated with sadness; and at the other “well-being,” “reptiles,” and “fantasy” but without a clear association with any emotions. In other words, the third dimension discriminates between a subjective component without a defined emotional attribution and an inanimate natural environment associated with sadness.

The fourth dimension explains 12.5% of the variance. At one extreme are situated “celestial bodies” and “fantasy,” associated with anger; and at the other extreme “built environment” and “woodland animals,” associated with disgust. This implies that the fourth dimension discriminates between an urban environment with animals associated with disgust and an imaginary environment associated with anger.

## Study 2

The mental maps generated by experience, which individuals draw on to understand reality, are not exempt from changes. A large body of literature claims that surprise arises as an emotional response to the perception of a strong stimulus, which, due to its perceptive magnitude and the necessity of accommodation it triggers, overwhelms our established mental maps (Lazarus, 1991). A positive correlation has been reported between the experience of awe, paying attention and preparing to confront unexpected occurrences (Marina, 2006; Faber and Hall, 2007). Furthermore, despite surprise being a complex emotion, it has been observed that children are able to recognize it from around the age of 6 years (Doan et al., 2018). The study of awe and surprise is increasingly forming part of research on environmental psychology (e.g., Lazarus, 1991; Keltner and Haidt, 2003; Shiota et al., 2007; Nasar and Cubukcu, 2011; Joye and Dewitte, 2016; Ballew and Omoto, 2018; Collado and Manrique, 2019).

### Methodology

#### Participants

Once we had excluded participants that had not presented their parents’ or guardians’ informed consent and two students with special educational needs, the sample comprised 39 children enrolled in the 5 years old pre-school classes at the Benjamín Palencia Infant and Primary School in Albacete, Spain, none of whom had participated in Study 1. Of these, 48.7% were girls and the mean age was 4.86 years (*SD* = 0.41).

#### Instrument and Procedure

To study the emotional attributions, the same procedure was followed as in Study 1, with images of natural environments, of which 56.7% were images of animals and insects and 43.3% were of landscapes. Specifically, and drawing on a first level classification, 20% of the images corresponded to the label of mammals, 10% birds, 6.7% fish, 3.3% reptiles, 16.7% arthropods, 3.3% cold landscapes, 10% forest landscapes, 10% landscapes with water, 10% dry landscapes, and 10% landscapes of flowers. As in the first study, the images were presented randomly.

**FIGURE 5:**
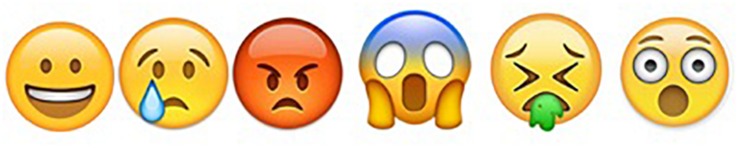
Emoticons from the response scale, emoticons reflecting happiness, sadness, anger, fear, disgust and surprise, in that order.

For the emotional attribution procedure, on this occasion, we used a response scale with six emoticons representing the previously used five basic emotions proposed by Ekman (1999), happiness, sadness, anger, fear and disgust, plus a sixth emotion, that of surprise (Figure 5), improving the scale from the study 1. This emotion was included due to its significance in studies on the environment, and because some of the children’s explanations in Study 1 could have been classified as expressions of surprise, but as it was not a choice among the five basic emotions, some of the children exhibited doubts when expressing a response.

The validity of the emoticons was once more submitted to inter-rater analysis. Two expert professionals from the field of preschool education completed a questionnaire in which they were required to rate three possible emoticons on a three-point scale (Appropriate = 1, Inappropriate = 2, Needs modifying = 3). Fleiss’ Kappa was.317, indicating a correct level of agreement (Dubé, 2008). The emoticons chosen were signaled correctly by the children in 92% of the cases.

We repeated the same contact, information and consent procedures with the school authorities and the children’s parents or guardians. The tests were administered individually in specially prepared rooms in the school during normal class time. The mean time taken to administer the test was around 30 min, but on this occasion, we recorded the children’s explanations for their attributions.

All the responses were recorded on an Excel spreadsheet. The data were analyzed using PSS24 for the descriptive analysis, χ^2^ (chi squared) and simple correspondence analysis, as well as the same procedure as study 1 to retain the number of dimensions and their interpretation; and we used Open Coding (Strauss and Corbin, 1994) for the qualitative analysis of explanations.

### Results

#### Emotional Attributions

To examine the emotional attributions, we conducted an analysis of frequency distribution on the emotions attributed to the images. The results revealed happiness was the emotion most frequently attributed (45.1%) and fear the second (23%).

With the assistance of experts in environmental sciences and biology, the images of natural environments were twice classified, according to their predominant elements and according to the food chain to which they belonged. The first classification distinguished between “vertebrates” “invertebrates” and “general landscape.” The frequency distribution of the emotional attributions according to these labels was significant (χ^2^ = 123.942; *p* < 0.001; η = 0.16). In the vertebrate category, happiness was the most frequently attributed emotion (40.6%), followed by fear (29.6%). In the invertebrate category, the most common emotion was fear (33%), followed by happiness (24.5%). Finally, in the landscape category, the most frequently attributed emotion was happiness (57.5%), followed by surprise (13.5%).

**FIGURE 6:**
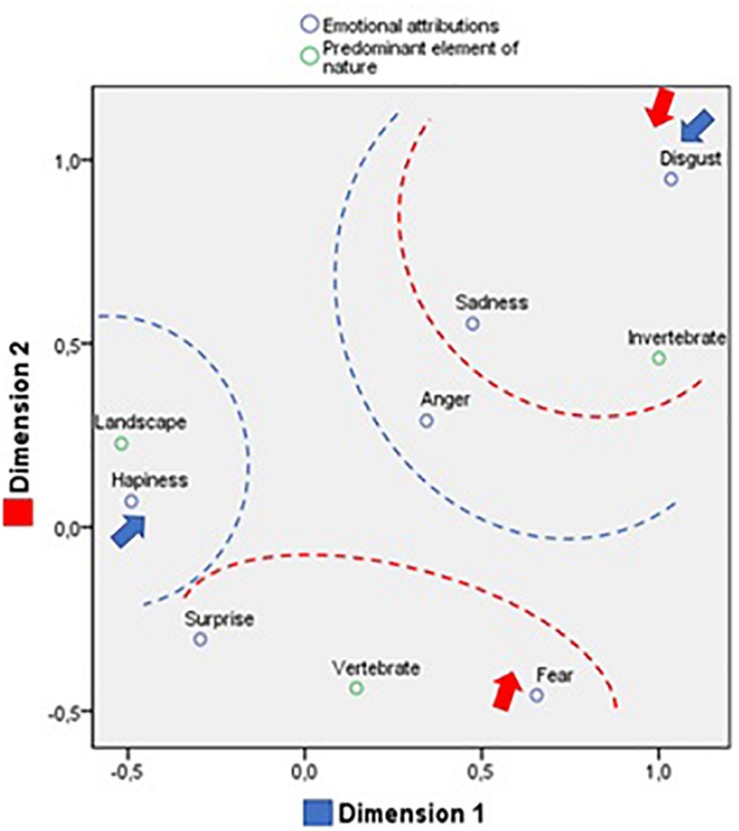
Correspondence analysis between emotional attribution and predominant elements of nature.

The correspondence analysis between the classification of the natural environment images according to their predominant natural elements (invertebrates, vertebrates and landscapes) and the emotional attributions revealed two dimensions (χ^2^ = 123.942; *p* = 0.000), which together explain 100% of the variance (see Figure 6 and Table 2).

**TABLE 2:** Symmetrical normalization of row and column points in the correspondence analysis between emotional attribution and predominant elements of nature.

	Mass			Inertia	Contribution				
					
		Score in dimension			Of point to inertia of dimension		Of dimension to inertia of point		
					
		1	2		1	2	1	2	Total
**Overview row points: predominant element of nature**									
Vertebrate	0.400	0.145	–0.438	0.013	0.029	0.571	0.193	0.807	1.0
Invertebrate	0.167	1.000	0.460	0.053	0.571	0.262	0.911	0.089	1.0
Landscape	0.433	–0.519	0.228	0.037	0.400	0.167	0.919	0.081	1.0
**Overview column points: emotional attributions**									
Happiness	0.453	–0.491	0.070	0.032	0.373	0.017	0.991	0.009	1.0
Sadness	0.098	0.474	0.554	0.011	0.076	0.224	0.614	0.386	1.0
Anger	0.054	0.344	0.290	0.002	0.022	0.034	0.754	0.246	1.0
Fear	0.228	0.655	–0.457	0.035	0.336	0.355	0.817	0.183	1.0
Disgust	0.043	1.035	0.948	0.018	0.156	0.284	0.721	0.279	1.0
Surprise	0.124	–0.296	–0.305	0.005	0.037	0.086	0.671	0.329	1.0

The first dimension explains 82.5% of the variance. At one extreme of the first dimension we find only happiness, although surprise falls near, together with the label “landscapes.” At the other extreme, are located the emotions of disgust, fear, sadness, and anger, which appear close to “invertebrates.” This means that the first dimensions distinguishes between landscapes eliciting positive emotions and living beings, which generate negatively emotions.

The second dimension explains 17.5%. At one extreme we find fear and surprise, together with the label “vertebrates,” while at the other, we find disgust and sadness, together with “invertebrates.” This suggests that the second dimensions distinguishes fear and surprise associated with vertebrates and disgust and sadness elicited by invertebrates.

The second classification of the images, according to food chain, differentiated between “consumers” (primary consumers, such as scorpion, centipede, bee, tarantula, fish, insect, or parrot), “hunters” (secondary consumers, such as a bat, penguin, snake, fox, leopard, vulture, shark, tiger, or sea lion) and “producers” (primary producers, corresponding to images of landscapes, flowers and fungi). The frequency distribution for the emotional attributions made according to these categories was significant (χ^2^ = 94.772; *p* < 0.001; η = 0.17). “Hunters” were mainly associated with fear (60%), “producers” with happiness (78.5%) and “consumers” showed similar percentages for happiness and fear (50%).

**FIGURE 7:**
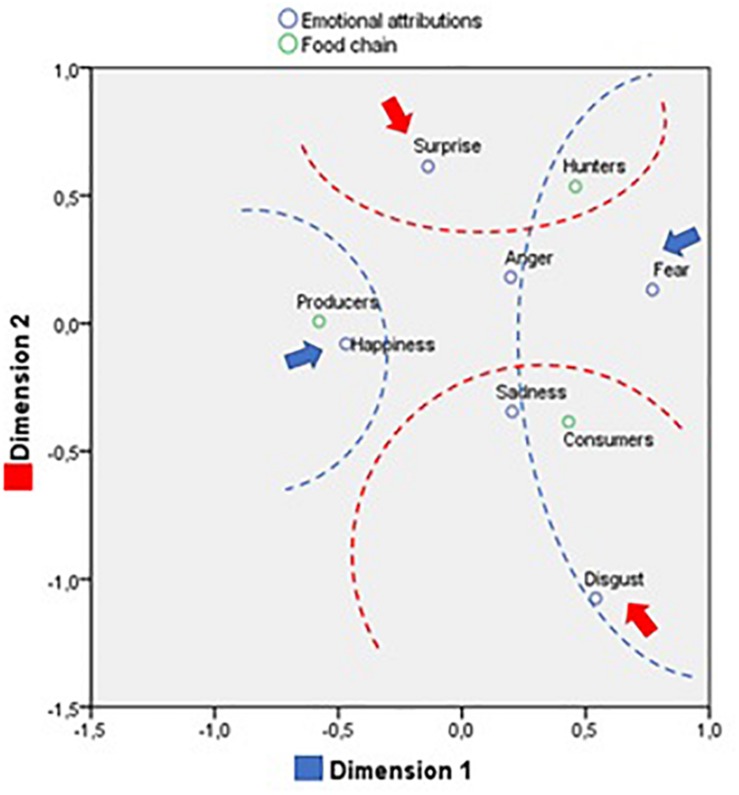
Correspondence analysis between emotional attribution and food chain.

The correspondence analysis for the classification of the natural environment images according to food chain and emotional attribution confirmed this trend, revealing two dimensions (χ^2^ = 94,772; *p* < 0.001), which together explain 100% of the variance (see Figure 7 and Table 3).

**TABLE 3:** Symmetrical normalization of row and column points in the correspondence analysis between emotional attribution and food chain.

	Mass			Inertia	Contribution				
					
		Score in dimension			Of point to inertia of dimension		Of dimension to inertia of point		
					
		1	2		1	2	1	2	Total
**Overview row points: emotional attributions**									
Happiness	0.453	–0.469	–0.080	0.026	0.389	0.025	0.987	0.013	1.0
Sadness	0.098	0.203	–0.346	0.002	0.016	0.101	0.431	0.569	1.0
Anger	0.054	0.197	0.180	0.001	0.008	0.015	0.724	0.276	1.0
Fear	0.228	0.770	0.131	0.035	0.529	0.034	0.987	0.013	1.0
Disgust	0.043	0.541	–1.076	0.009	0.049	0.423	0.357	0.643	1.0
Surprise	0.124	–0.137	0.614	0.006	0.009	0.402	0.099	0.901	1.0
**Overview column points: food chain**									
Consumers	0.333	0.431	–0.385	0.022	0.242	0.425	0.734	0.266	1.0
Hunters	0.233	0.459	0.535	0.020	0.192	0.575	0.618	0.382	1.0
Producers	0.433	–0.578	0.008	0.037	0.566	0.000	1.000	0.000	1.0

The first dimension explains 82.9%. At one extreme is situated happiness together with the “producers” category, while at the other extreme, we find fear and disgust, with “hunters” and “consumers.” This suggests the first dimensions distinguishes between landscapes associated with happiness and insects and certain animals associated with a negative emotional valence.

The second dimension explains 17.1%. At one extreme are situated the emotions of disgust and sadness, together with the category of “consumers,” while at the other, we find only surprise close to the “hunters” category. Thus, we can observe a distinction between animals causing a certain degree of repulsion and hunters that produce surprise.

#### Explanations for the Emotional Attributions

We performed a qualitative lexicographic analysis (Open Coding) on the explanations provided by the participants for their attributions to each of the 30 natural environment images, where the units of analysis were the entire sentences so as to avoid loss of semantic quality (Strauss and Corbin, 1994). A corpus of 2695 sentences was obtained, of which 1501 referred to the natural environment. We then drew up 21 categories to define the classification and facilitate the analysis. We then described the frequency of the emotional attributions made to each one (Table 4).

**TABLE 4:** Descriptive and frequency analysis of the 21 explanatory labels of the reasons for the emotional attributions given to the natural environments images.

Categories	Happiness	Sadness	Anger	Fear	Disgust	Surprise	Total	Percentages (%)
Activity	42	8	0	4	0	36	90	6.00
Association	26	0	2	18	1	21	68	4.53
Beauty	35	0	0	0	0	27	62	4.13
Characteristic	32	0	6	24	9	17	88	5.86
Quality	13	3	1	8	0	11	36	2.40
Taking care	11	8	0	0	0	9	28	1.87
Harm	3	71	30	15	6	11	272	18.12
Emotion	18	20	8	13	9	3	71	4.73
Season	2	0	0	0	0	1	3	0.20
Liking	320	0	0	0	0	39	367	24.45
Insects	0	2	0	6	28	0	36	2.40
Meteorology	7	2	2	5	1	3	20	1.33
No beauty	0	1	0	4	18	0	23	1.53
Disliking	0	11	25	35	20	2	93	6.20
No danger	12	0	0	0	0	6	18	1.20
Novelty	17	1	0	0	0	47	63	4.20
Danger	0	9	13	52	0	6	80	5.33
Worry	2	4	1	5	0	2	14	0.93
Overcoming fear	2	0	0	0	0	0	2	0.13
Tautology	8	2	1	4	1	6	22	1.47
Experience	34	2	0	3	1	5	45	3.00

The most frequent explanations referred to liking (24.45%) and harm (18.12%). Liking was more frequently associated with happiness (21.31%) and surprise (2.59%). Within this category, we found content that better explained why the children had made this attribution, referring to their liking of animals, vegetation, landscape, water, etc. Examples included: *“I like it because the trees are really big”; “because I like fish”; “because I love nature and all the bugs.”*

After liking, activity (6%), beauty (4.53%), and experience (3%) are the labels most frequently associated with the positively valenced emotional attributions (happiness and surprise).

Harm is the most common category in the emotional attributions of fear (10.06%). When harm was associated with fear or sadness, the explanations alluded to internal harm, directly suffered by the children. Examples include: *“it scares me because it might sting me and I’d cry”*; *“because it could eat me”*; or *“because it bites.”*

The categories of overcoming fear (0.13%), season (0.20%), and worry (0.93%) were the least frequently mentioned. Nonetheless, overcoming fear was associated with happiness, season with happiness and surprise, and worry with all the emotions except disgust.

## Discussion and Conclusion

Following the biophilia theory (Wilson, 1984), the relationship between human beings and the environment from the perspective of connectedness to nature (Mayer and Frantz, 2004) presupposes a connection based on an innate, positive predisposition. There is an extensive body of literature drawing on empirical studies on the benefits for well-being of contact with nature, measured in terms of positive physiological indicators of feelings of restoration. However, despite the evidence, the literature tends to ignore the possible adaptive function of negative emotional reactions, as part of a construct we might call biophobia. This is arguably due to a stigmatization of the negative hedonic tone and the methodological difficulties of studying the phenomenon in child populations.

The present study provides a valid measurement procedure to study the emotional attributions of 5 years old in response to images of natural environments. The inter-rater validation, the patterns of the participants’ correct responses for the trial images, and the results obtained in our two studies with different samples appear to confirm the validity of the procedure. The participants recognized and attributed emotions to the images in a spontaneous and immediate manner, and the initial instructions for the procedure were rapidly assimilated. The six-emotion protocol is the most appropriate, given that, in Study 1, the participants spontaneously identified surprise among the emotions generated by the images, while, in addition, this emotion is corroborated in the literature (Gosselin and Simard, 1999; Liu and Fang, 2007; Sauter et al., 2015).

Regarding the concept of “nature” as mentioned by the children and addressed in Study 1, similarities were found with the findings of previous studies (Collado et al., 2016). For example, the distinction appears between natural and non-natural elements, a description extended in the present study by the use of categories, such as “vegetation,” “natural environment,” and different types of “animals.” In addition, the human-nature interaction and emotional experiences associated with nature were identified in expressions used by the children in reference to the concept, and which can be observed in labels such as “human beings,” “esthetic emotion,” “well-being,” or “natural processes.”

Coinciding with the findings of previous studies (Fägerstam, 2012; Gilberston, 2012), happiness is the emotion most frequently attributed to the images of natural environments (landscapes). The higher frequency of attributions of happiness may be the result of feelings of agreeableness generated by the recall of pervious experiences in similar settings, which reinforces, as posited in the theory of connectedness to nature, the importance of contact with nature for the development of positive emotional traits (Mayer and Frantz, 2004; Schultz and Tabanico, 2007; Mayer et al., 2009; Olivos and Clayton, 2017; Mena et al., 2020).

Nonetheless, fear, as a response to unpleasant or threatening images, such as those of factories, traffic jams, sharks or snakes, is the second most frequently attributed emotion. The results of the correspondence analysis between the concept of nature and the emotional attributions are in a similar line, as they suggest that children associate nature with happiness, when the images refer to landscapes, places with other people or animals that might be thought of as pets, but they also see nature as a threatening, hostile environment, related to emotions of disgust and fear.

The second study allowed us to confirm that happiness is the emotion most commonly associated with natural environments, and fear the second. Detailed analysis of the correspondences between the emotional attributions and the natural environment revealed relationships between fear, sadness and anger, mainly in response to images of wild animals, insects or natural landscapes that might involve a certain level of danger.

The classification of the images by both predominant elements and by food chain also revealed similar findings. The results of the analysis according to predominant elements suggest the attributions can be interpreted across two axes, where surprise plays a dual role. That is, the first axis would run from awe (happiness and surprise) attributed to landscapes, to threat or harm (rest of negatively valenced emotions) associated with living beings. The second axis would distinguish between living beings, associating a state of alert (surprise and fear) in response to vertebrates and a reaction to harm (disgust, sadness and anger) associated with invertebrates. The categorization of the images by food chain suggests a distinction between animals that generate a certain feeling of repulsion (e.g., bats and snakes) and hunters, which elicit surprise, associated here with the activation of a state of alert.

Study 2 shows the importance of including surprise as a possible emotional response in children, despite being a complex emotion that may, on some occasions, be accompanied by fear and on others by happiness. It has been reported that a reaction of surprise or awe is primarily a response to positive stimuli (Keltner and Haidt, 2003). However, it has also been shown that in the face of sudden, unexpected situations, individuals focus and prepare themselves for unanticipated scenarios (Marina, 2006), an example being the reflex to fight or flee, which facilitates a rapid but short-term cognitive and physiological activity that displaces other emotions. Studies in emotional psychology have shown that fear and surprise trigger initially similar facial expressions but a few seconds later differences emerge when either fear or surprise is fully expressed (Jack et al., 2014). Although happiness and surprise both appear in response to positively valenced stimuli, the difference lies in that stimuli that generate happiness induce self-focused attention, while awe focuses attention on the perception and interpretation of a situation in relation to oneself (Salovey, 1992; Silvia and Abele, 2002), facilitating other adaptive associations.

The findings of our analyses of emotional explanations provides information in favor of our interpretations. Expressions of happiness are typically associated with explanations based on the esthetic value of natural environments and their elements, or environments that present a challenge where fear must be overcome. Instead negative emotions appear associated with a perception of nature as a source of displeasure, danger or physical harm. Finally, such attributions also draw on a series of disagreeable natural elements and environments associated with disgust.

Thus, in light of our findings, two main conclusions may be drawn. The first is a tripartite hypothesis based on the valences of the attributed emotions, where we observe that potentially threatening animals are associated with surprise and fear, insects and certain consumers are associated with disgust, and other elements of nature identified as producers or landscapes are associated with happiness. The second conclusion is that these emotional attributions coincide with the findings of other authors on the importance of biophobic content in connectedness to nature (Orr, 1994; Freire, 2011), whereby negative emotions offer a valuable adaptive function.

Finally, the present study provides empirical evidence and procedures for the study of environmental psychology in 5 years old. The natural environment is regarded as key for the successful development of coming generations. Hence, it is of importance to reflect on the link between today’s children and the environment, to address questions on their concept of nature, and to develop emotional training with regard to the natural environment in an educational context where positive psychology and emotional intelligence have enabled a partial perspective on nature.

## Data Availability Statement

The datasets generated for this study are available on request to the corresponding author.

## Ethics Statement

The study was carried out under the ethical conditions of the UCLM Department of Psychology and the University Vice-Rectorate of Research, which is requested for not an experimental study; authorization was requested from those responsible for the participating institutions, as well as the informed consent of the parents. Written informed consent to participate in this study was provided by the participants’ legal guardian/next of kin.

## Author Contributions

PO-J: substantial contributions to the conception or design of the work; analysis and interpretation of data for the work; drafting the work; providing approval for publication of the content. RS-F: substantial contributions to the acquisition, analysis or interpretation of data for the work; drafting the work; providing approval for publication of the content. CR-P: substantial contributions to the acquisition, analysis or interpretation of data for the work; providing approval for publication of the content. BF-G: substantial contributions to the acquisition of data for the work; providing approval for publication of the content.

## Conflict of Interest

The authors declare that the research was conducted in the absence of any commercial or financial relationships that could be construed as a potential conflict of interest.
